# Effects of Wollastonite Fiber and Styrene–Butadiene Latex Polymer on the Long-Term Durability of Cement-Based Repair Materials

**DOI:** 10.3390/ma15155433

**Published:** 2022-08-07

**Authors:** Yeon Jae Choo, Geon Hee Lee, Su-Jin Lee, Chan-Gi Park

**Affiliations:** 1 Department of Agricultural Engineering, Kongju National University, Yesan 143-701, Korea; 2 Department of Architectural Engineering, Keimyung University, Daegu 42601, Korea; 3 Department of Regional Construction Engineering, Kongju National University, Yesan 32439, Korea

**Keywords:** durability, latex polymer, repair materials, ultra-rapid hardening cement, wollastonite mineral fiber

## Abstract

Concrete structures are constructed in various geographical environments and climates, and frequently fail to fulfill their original functions over time due to issues such as aging and damage. Research on concrete structure repair materials is being conducted to solve these problems. This study evaluated the durability of a repair material composed of ultra-rapid hardening cement, styrene–butadiene (SB) latex polymer, and wollastonite mineral fiber. The performance targets were as follows: compressive strength of 20 MPa at 1 day of age and 45 MPa at 28 days of age, chloride ion charge passed of less than 1000 Coulombs, carbonation depth of 20 mm or less, and resistance to repeated freezing and thawing (relative dynamic modulus of elasticity) of 80% or more. The ultra-rapid hardening cement:silica sand ratio of 1:1.5 was the experimental variable, and the unit weight of each material in the mix proportion was determined to satisfy the flow requirement of 200 ± 5 mm. This flow ensured sufficient fluidity for spraying, which is the most widely used method for applying repair material. Wollastonite mineral fiber and SB latex polymer were added at 3% and 5% of the unit weight of the binder, respectively. The mechanical property of the repair material was evaluated through compressive strength, and durability was evaluated through chloride ion penetration, alkali resistance, resistance to carbonation, water absorption, and repeated freezing and thawing tests. The compressive strength satisfied both target values, regardless of the addition of SB latex polymer and wollastonite mineral fiber. The chloride ion penetration test, which was used as an indicator of durability, showed that mixtures without SB latex and wollastonite mineral fiber were not satisfied the target charge passed of 1000 Coulombs, while mixtures with latex and mineral fiber reached the target value. Notably, the co-addition of latex and wollastonite fiber showed the highest resistance to chloride ion penetration, alkali ion, carbonation, repeated freezing and thawing, and the least absorption. The results confirmed that the durability of the repair material based on ultra-rapid hardening cement was most effectively improved by the co-addition of SB latex polymer and wollastonite mineral fiber.

## 1. Introduction

Research concerning the durability of concrete has established that causes of concrete performance degradation over time include various external environmental factors, such as carbonation, salt damage, chemical erosion, cracks, and freezing damage, and internal factors such as the alkali-aggregate reaction [1,2,3]. Research on repair materials for concrete structures is being conducted to solve these issues. Considerable time and money are required to build civil engineering facilities. Hence, it is preferable to increase the useable lifetime of the structure via repair and reinforcement rather than construct a new structure, provided that the safety of the structure is not compromised [4]. Various types of polymer-modified repair materials are used for concrete. The excellent mechanical properties and durability of these materials are due to the high performance of the polymer [5]. In addition, the spray method, which is typically used to repair concrete structures, facilitates construction and shortens the construction time. However, there is a strong likelihood that cracks will appear after spraying, which may increase permeability and decrease durability. Therefore, the polymer repair material used for the spray method must minimize the possibility of crack occurrence. As a cement-based inorganic material, ultra-rapid hardening cement is often used to ensure initial strength. Although the initial rapid increase in the heat of hydration of this type of cement increases the initial strength, it also increases the risk of cracks inside the repair material [6]. The resulting increased permeability shortens the re-repair period of the concrete structure [6]. Therefore, it is necessary to apply the material that has crack-resistant. Generally, reinforcing fiber is used to improve the crack control of repair material. However, the absence of coarse aggregate in the repair material makes it difficult to uniformly distribute the reinforcing fiber, thereby increasing the risk of nozzle clogging during spraying due to fiber balling. The reinforcing fiber must not make the repair material prone to clogging nor detrimentally affect the fluidity. Wollastonite mineral fiber has a density of about 2.9 g/cm^3^, a chemical composition similar to that of cement-based binder materials, and reinforcing properties [7]. It was considered that wollastonite mineral fiber in a cement-based material would provide the required dispersibility and also solve the fiber clumping issue. It could also provide crack control and mitigate fiber balling in the repair material. Recent research has shown that wollastonite mineral fiber improves crack control and flexural properties when added to cement-based materials as a reinforcement [8,9]. Styrene–butadiene (SB) latex polymer is typically added to the repair material to provide sufficient fluidity when spraying, and its improved adhesion can reduce rebound during spraying [10]. In addition, SB latex polymer improves permeability resistance via film formation, which improves durability [11]. Herein, wollastonite mineral fiber was used to improve crack resistance and durability, and SB latex polymer to ensure fluidity, facilitate construction, and improve durability. The co-addition of SB latex polymer and wollastonite mineral fiber was expected to control nozzle clogging and improve construction ability and durability via crack control. This research identified the mixing ratio that satisfied the fluidity and target mechanical properties required for spraying a repair material composed of ultra-rapid hardening cement, SB latex polymer, and wollastonite mineral fiber. In other words, the proper amounts of studied materials; the wollastonite mineral fiber, SB latex polymer, ultra-rapid hardening cement, and silica sand were determined to satisfy the fluidity requirement of the spray method. The strength of the repair material was assessed according to its compressive strength, and its durability was assessed based on its chloride ion penetration resistance, alkali resistance, resistance to carbonation, water absorption, and resistance to repeated freezing and thawing.

## 2. Experimental Plan

### 2.1. Materials

The chemical composition of the ultra-rapid hardening cement (Joog-Ang Polytech Co., Ltd., Gyeongsangnam-do, Korea) is given in Table 1. Silica sand is used as the fine aggregate and has a density of 2.62 g/cm^3^ and a fineness modulus of 1.95 (Table 2). Wollastonite mineral fiber has a density of 2.9 g/cm^3^ and aspect ratio ranging from 3 to 20; the main physical properties are listed in Table 3. The properties of the SB latex polymer (Joog-Ang Polytech Co., Ltd., Gyeongsangnam-do, Korea) are listed in Table 4; its solid content is about 49 wt.%.

### 2.2. Mix Proportion

This study was conducted to evaluate the effects of wollastonite fibers and SB latex polymers on the long-term durability of cement-based repair materials. The studied mix proportions are listed in Table 5. The target values of each property are as follows: compressive strength of 20 MPa at 1 day and 45 MPa at 28 days, chloride ion charge passed of less than 1000 Coulombs, carbonation depth of 20 mm or less, and resistance to repeated freezing and thawing (relative dynamic modulus of elasticity) of 80% or more. A cement: silica sand ratio of 1:1.5 was used. The target flow was 200 ± 5 mm, which is determined by KS F 2476. The unit weight of each material in the mix proportion required to provide sufficient fluidity for spray application was determined; the results are presented in Table 5. Wollastonite mineral fiber and latex were incorporated at 3% and 5% of the unit weight of the binder, respectively.

### 2.3. Experimental Methods

#### 2.3.1. Compressive Strength Test

In this study, the effects of wollastonite fibers and SB latex polymers on the compressive strength of cementitious repair materials were evaluated. The compressive strength of the repair material was determined according to the KS L 2476. The sized 50 × 50 × 50 mm cubic specimen was prepared and initially cured at a constant temperature of 23 ± 2 °C and 65 ± 5% relative humidity (RH) for 24 h. Compressive strength test specimens were manufactured by nine specimens each for each mixture, and compressive strength tests were carried out by three specimens each for each curing days (1, 7, and 28 days). After the initial curing, the form was removed and water curing was performed at a constant temperature of 23 ± 2 °C for 27 days. For evaluating the compressive strength, three specimens were manufactured and loaded at 2400 ± 200 N/s. The compressive strength test result was presented as an average value after testing three specimens for each mixture.

#### 2.3.2. Resistance to Chloride Ion Penetration Test

**Figure 1:**
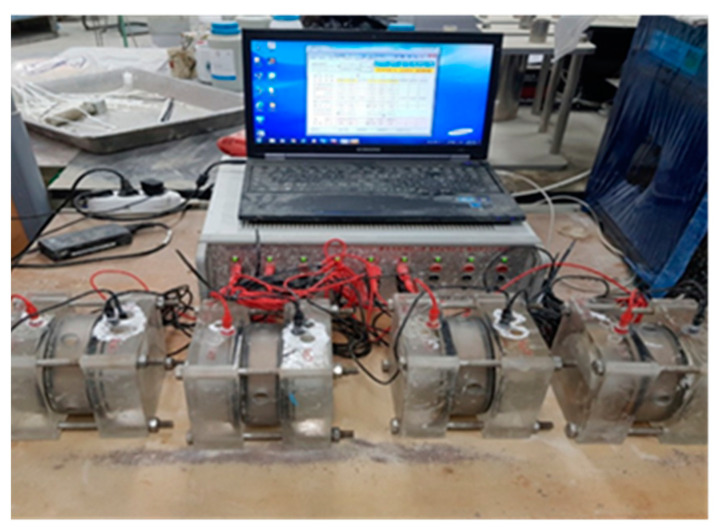
Photo of chloride ion penetration test setup.

In this study, the effect of wollastonite fiber and SB latex polymer on improving the water permeability of cement-based repair materials was evaluated. The water permeability resistance was evaluated by the chlorine ion permeation test, which is an indirect water permeation test method for concrete. The resistance to chloride ion penetration was measured according to ASTM C1202. Cylindrical specimens with dimensions of Ø100 × 200 mm were prepared, cured in the same condition as specimens for compressive strength, and cut into 50 mm thick slices. Trapped air was removed by placing a specimen into a desiccator and applying a vacuum for 3 h, after which sufficient water was added to completely submerge the specimen and the system was evacuated for another 1 h. Then, the vacuum pump was shut off and the specimen was left to soak in the water for 18 ± 2 h [12]. The specimen was then placed in the measuring cell and the current was recorded for 6 h. The cathode of the cell was filled with 3% NaCl solution and the anode with 0.3 N NaOH solution; the electrodes were connected to a power supply providing DC 60 ± 0.1 V [3]. The target charge passed was 1000 Coulombs or less. Figure 1 shows the experimental setup. The chloride ion penetration test result was presented as an average value after testing three specimens for each mixture.

#### 2.3.3. Alkali Resistance Test

**Figure 2:**
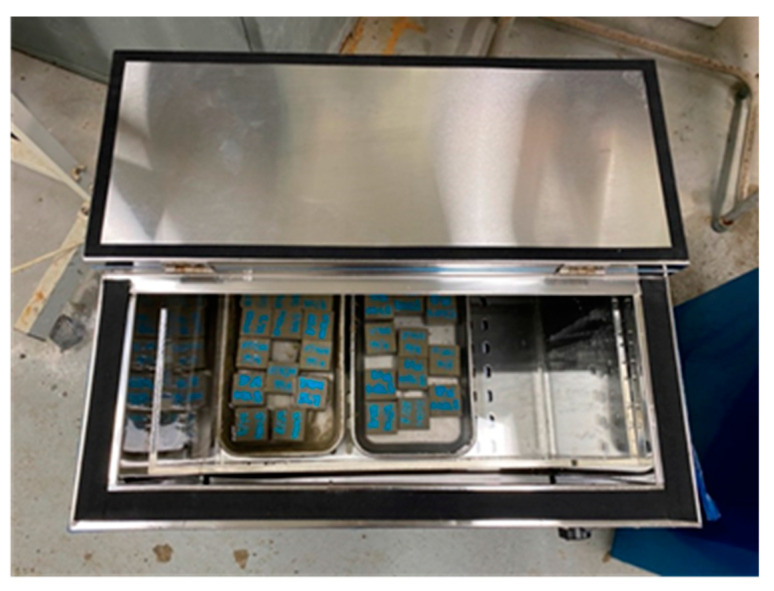
Photo of alkali resistance test.

Concrete is strongly alkaline due to the hydration reaction between cement and water. In this study, the effect of the addition of wollastonite fiber and SB latex polymer on the strength characteristics of cement-based repair materials when exposed to an alkaline environment was evaluated. The alkali resistance test was performed to confirm the change in the strength of the mortar in the presence of alkali [13]. A concrete repair material should keep enough strength retention while soaking in saturated hydroxide solution for 28 days (Figure 2). The test was carried out according to KS F 4042. Three cubes sized 50 × 50 × 50 mm were cured for 28 days and then soaked in saturated calcium hydroxide solution at 50 ± 2 °C for additional 28 days. Then, the specimens were taken out of the solution, wrapped with a wet cloth, and cooled at room temperature for 1 day. The compressive strength was then measured. The alkali resistance test result was presented as an average value after testing three specimens for each mixture.

#### 2.3.4. Resistance to Carbonation Test

**Figure 3:**
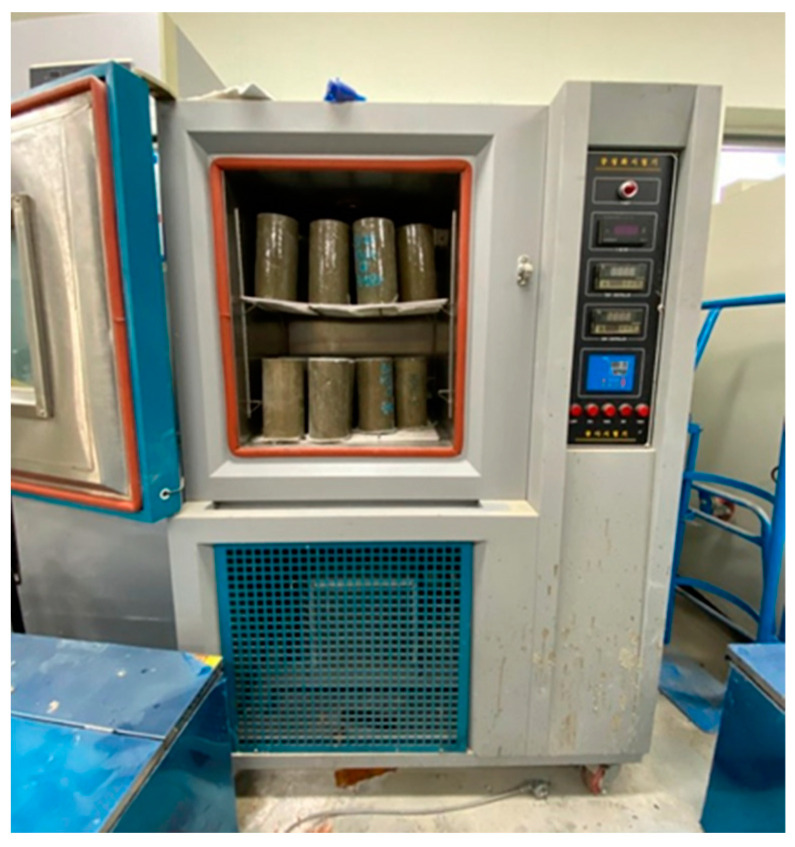
Photo of neutralization test device.

The carbonation of concrete is a process through which the alkalinity of the concrete is lost via the action of carbonic acid, acidic gases, and salts present in air or water. Carbonation is one of the major causes of degraded concrete durability, such that the resistance to carbonation is an important performance metric for repair material [14]. In this study, the effect of the addition of wollastonite fiber and SB latex polymer on the improvement of carbonation resistance of cement-based repair materials was evaluated. In the test, the depth where carbonation occurs is measured after the repair material has been placed in the carbonation-accelerating environment. Three cylindrical test specimens with dimensions of Ø100 × 200 mm were prepared and cured for 28 days at 20 ± 2 °C and 65 ± 10% RH (Figure 3). For the test, epoxy was applied to all surfaces except the top surface. The carbonation depth was measured after exposing the specimens to the 5% carbon dioxide concentration in the test chamber for 28 days, as follows: 1% phenolphthalein solution was sprayed on a cut surface of a specimen and the color change in the surface was observed. The length from the edge to where the color is no longer red is referred to as the carbonized part. The depths at three locations on each side edge of the carbonized part were measured using a Vernier caliper, for a total of six measurements. The resistance to carbonation was considered excellent if the depth at which carbonation proceeded was 20 mm or less. The carbonation test result was presented as an average value after testing three specimens for each mixture.

#### 2.3.5. Water Absorption Test

Harmful materials can permeate into a concrete structure through an exposed surface, thereby degrading the durability of the structure [15]. The water absorption of the repair material is to assess the permeability of water into the concrete surface [15]. In this study, the effect of the addition of wollastonite fibers and SB latex polymer on the water absorption properties of cement-based repair materials was evaluated. The absorption test was carried out according to KS F 2476. Three prismatic specimens sized 40 × 40 × 160 mm were prepared, cured for 28 days, dried in an oven at 80 ± 2 °C for 48 h, cooled in a desiccator for 1 day, and weighed. This weight is referred to as the dry weight (*W*_0_). Then, the specimen was immersed in clean water at 20 ± 2 °C for 48 h, after which time the specimen was taken out of the water, the surface of the wet specimen was quickly wiped with a cloth, and the weight was measured. This weight is referred to as the after-absorption weight (*W*_1_). The absorption rate was calculated as follows.

$$\mathrm{Absorption}\;\mathrm{rate}\;(\%)=\frac{W_{1}-W_{0}}{W_{0}}100\;(\%)$$

The water absorption test result was presented as an average value after testing three specimens for each mixture.

#### 2.3.6. Repeated Freezing and Thawing Test

**Figure 4:**
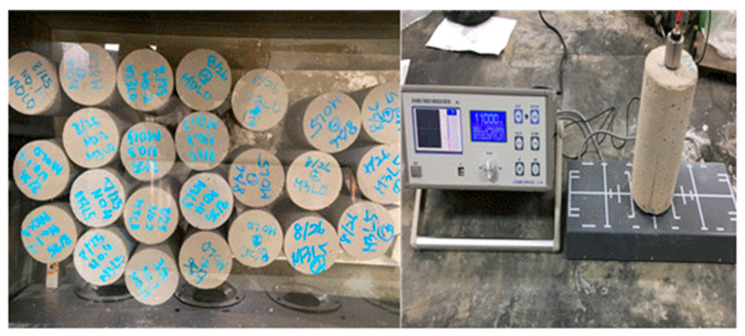
Photo of repeated freezing and thawing cycle test setup.

In this study, the effect of the addition of wollastonite fibers and SB latex polymer on the durability of cement-based repair materials exposed to repeated freezing–thawing environments was evaluated. The repeated freezing and thawing test was carried out according to KS F 2456 (type A: underwater freezing and underwater thawing), to confirm the durability performance of the repair material under a repetitive freezing and thawing environment, as occurs in winter. The freezing and thawing cycle consisted of lowering the temperature of the specimen from 4 to –18 °C, followed by increasing it from –18 to 4 °C, within 2–4 h [16]. Three cylindrical test specimens sized Ø100 × 200 mm were prepared, cured for 14 days, and then placed in the conditioned chamber. The relative dynamic modulus of elasticity was measured every 30 cycles according to KS F 2437. The repeated freezing and thawing test result was presented as an average value of three specimens for each mixture. Figure 4 shows the test setup.

## 3. Test Results

### 3.1. Compressive Strength Results

**Figure 5:**
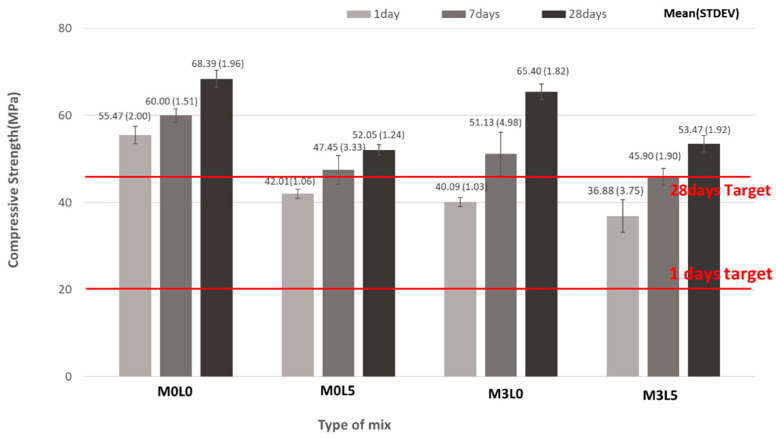
Compressive strength test result.

Figure 5 presents the compressive strength test results. All of the mixtures satisfied the target compressive strength of 20 MPa at 1 day of age and 45 MPa at 28 days of age, i.e., all mixes have exceeded the target compressive strength. The compressive strength of the M0L5 and M3L5 mixes, which contained SB latex polymer, was lower than that of the wollastonite- and latex-free M0L0 mix because the presence of the SB latex delayed the hydration reaction of the ultra-rapid hardening cement, thereby decreasing the compressive strength of the repair material [9,17]. Compared with the M3L5 mix, which contained both wollastonite fiber and latex, the compressive strength of the M0L5 mix was lower at 1 and 7 curing days, but similar at 28 curing days. This result is because the amount of ultra-rapid hardening cement was smaller in the wollastonite-containing M3L5 mix, which affects the initial compressive strength. However, the similar strength at 28 days suggests that wollastonite may offset the earlier reduction in compressive strength in the long term. Nevertheless, the target compressive strength of 20 MPa at 1 day and 45 MPa at 28 days were met by the M3L5 mix, indicating its suitability as a repair material.

### 3.2. Resistance to Chloride Ion Penetration Results

**Figure 6:**
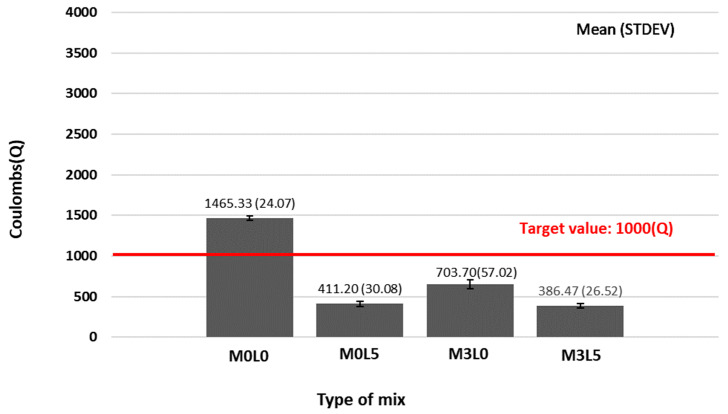
Chloride ion penetration test results.

Figure 6 presents the resistance to chloride ion penetration test results. The target value of charge passed was achieved by almost every mixture; 1000 Coulombs or less (excluding the M0L0 mix). Notably, the M3L5 mix has the lowest chloride ion penetration due to the densification of the internal structure of the concrete by wollastonite mineral fiber and SB latex polymer film. Their co-addition performed better than the fiber- and latex-only cases.

### 3.3. Alkali Resistance Results

**Figure 7:**
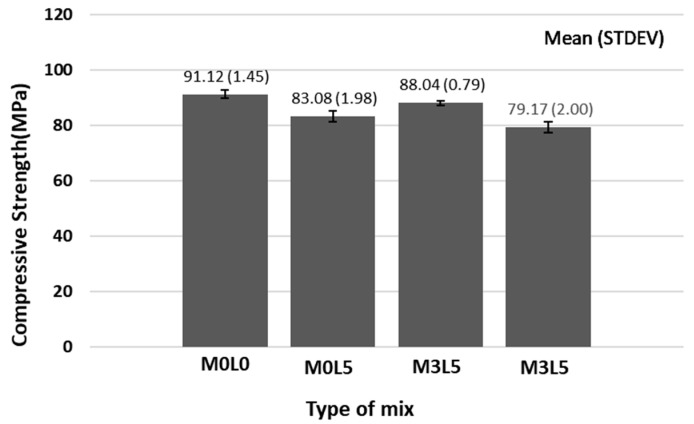
Alkali resistance test result.

Figure 7 presents the alkali resistance results of the studied repair materials by comparing the compressive strength of the repair material after immersion in saturated calcium hydroxide solution for 28 days. The compressive strength of all mixtures was about 80 MPa, and there was no apparent influence of the test solution. The trend was similar to that of normal compressive strength at 28 days, i.e., the compressive strength of the mixes containing SB latex polymer was lower, and that of the mix containing only wollastonite mineral fiber was similar to that of the M0L0 mix. The strength of the mix containing both SB latex polymer and wollastonite mineral fiber was not significantly different from that made with only latex. In addition, the 28-day compressive strength increased after immersion in alkaline solution because the effect on the alkali solution due to the formation of a film of latex was insignificant and the enhancing effect of long-term compressive strength due to the addition of latex. This apparent lack of significant effect of the alkaline test solution is an indicator of the excellent durability of the concrete repair material containing wollastonite mineral fiber and SB latex.

### 3.4. Resistance to Carbonation Results

**Figure 8:**
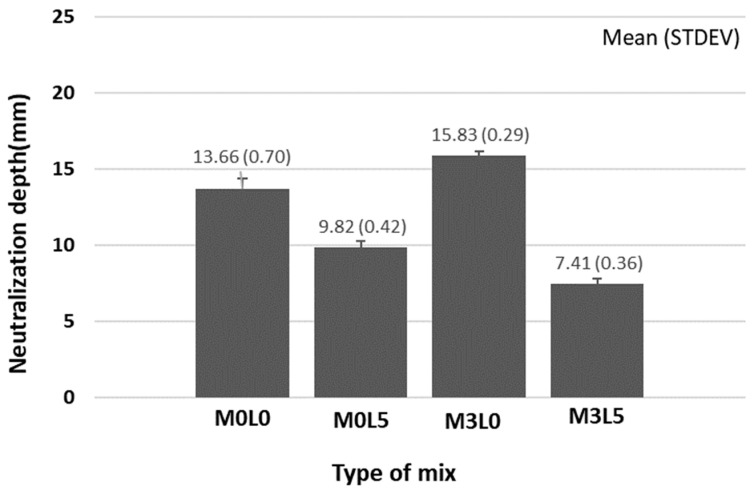
Neutralization resistance test result.

**Figure 9:**
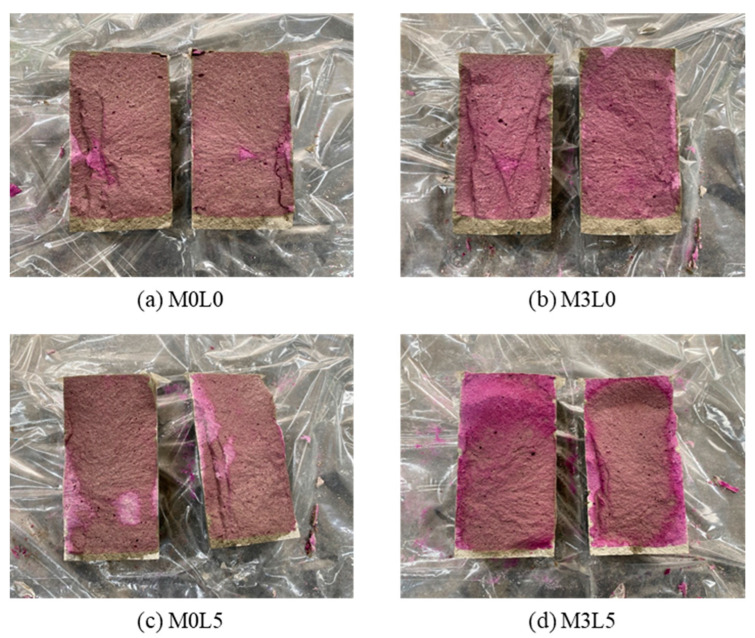
Photo of measuring the depth of neutralization.

Figure 8 and Figure 9 present the resistance to carbonation test results. All of the mixtures satisfied the permeation depth requirement of 20 mm or less. Notably, the M3L5 mix that contained both wollastonite and latex had the smallest carbonation depth, at less than 10 mm. The fine wollastonite fibers and latex film occupied and sealed inner pores. Mixtures containing latex had smaller carbonation depths than those containing wollastonite mineral fiber. This was attributed to SB latex polymer film formation that prevented CO_2_ gas from permeating. The higher density of the concrete containing both wollastonite mineral fiber and SB latex polymer reduced permeability.

### 3.5. Water Absorption Results

**Figure 10:**
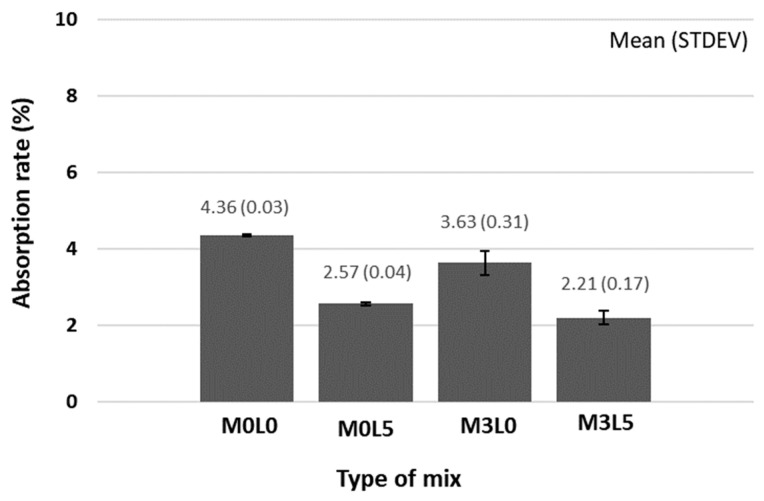
Water absorption test result.

Figure 10 presents the water absorption test results. Due to film formation, the mix containing only SB latex polymer had lower absorption compared with that made with only wollastonite mineral fiber. The addition of wollastonite mineral fiber provided crack control and densified the structure, which resulted in lower absorption. Notably, the mix containing both SB latex polymer and wollastonite mineral fiber was the least absorptive. Generally, osmotic pressure drives water permeation in cement paste, and it is likely that the fine wollastonite fibers and latex film formation sealed pores and prevented water from penetrating the cured repair material. Simultaneous incorporation of wollastonite mineral fiber and SB latex polymer was thus more beneficial than adding either separately.

### 3.6. Repetitive Freezing and Thawing Results

**Figure 11:**
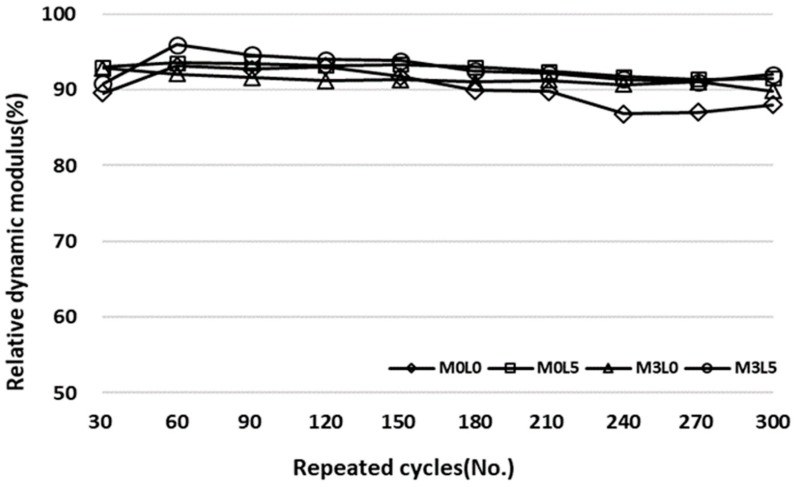
Repeated freezing and thawing cycles test results.

Figure 11 presents the repeated freezing and thawing test results. All of the mixes satisfied the relative dynamic modulus of elasticity requirement of 80% or more. The wollastonite- and latex-free M0L0 mix had a relative dynamic modulus of elasticity of about 88%, which compares with about 90% for the repair material containing 3% of wollastonite mineral fiber, indicating a slight improvement in the freezing and thawing test. Co-addition of SB latex polymer and wollastonite mineral fiber also led to some improvement.

## 4. Conclusions

Compressive strength and durability evaluations were carried out to confirm the properties of the ultra-rapid hardening cement-based repair material containing SB latex polymer and wollastonite mineral fiber as a repair material for concrete structures. Durability was assessed through the resistance to chloride ion penetration, alkali resistance, and carbonation, as well as water absorption and repeated freezing and thawing. The conclusions of the study are as follows:

- Every mixture satisfied the target compressive strengths of 20 MPa at 1 day and 45 MPa at 28 days. Adding SB latex polymer and wollastonite mineral fiber had adverse effects on the compressive strength, but the strength improved with time.
- Resistance to chloride ion penetration test confirmed that all mixes other than the wollastonite- and latex-free mix (M0L0) met the requirement of 1000 Coulombs or less. The wollastonite mineral fiber and SB latex polymer, used individually or together, decreased the permeability of the repair material. None of the mixes were affected by the saturated calcium hydroxide solution used in the alkali resistance test. The compressive strengths after exposure to the test solution were similar to those after 28 days. Exposure to alkaline solution did not significantly affect the 28-day compressive strength.
- The lowest compressive strengths were observed for mixes containing SB latex polymer, and the compressive strength of the M3L0 mix containing only wollastonite mineral fiber was similar to that of the wollastonite- and latex-free mix (M0L0).
- The presence of SB latex polymer improved resistance to carbonation through the formation of a latex film. The mix containing both wollastonite mineral fiber and SB latex polymer has excellent carbonation resistance (less than 10 mm).
- The addition of SB latex polymer or wollastonite mineral fiber decreased water absorption, and their combination exerted an even stronger effect. This was attributed to the fine wollastonite fibers and latex film filling and sealing the pores, thereby preventing water from permeating the structure.
- In the resistance to freezing and thawing test, all mixes met the requirement of a relative dynamic modulus of elasticity of 80% or more. The value of 88% obtained for the wollastonite- and latex-free M0L0 mix was improved to 90% or more when both components were present. Co-addition was more effective for improving the resistance to freezing and thawing due to the formation of a latex film, densification, and improved crack control of the structure by wollastonite.
- Co-addition of SB latex polymer and wollastonite mineral fiber was the most effective for improving the durability of ultra-rapid hardening cement-based repair material.

## Figures and Tables

**Table 1:** Chemical composition of ultra-rapid hardening cements.

Chemical Composition (%)							Blaine (cm^2^/g)	Density (g/mm^3^)
SiO_2_	Al2O_3_	Fe_2_O_3_	CaO	MgO	K_2_O	SO_3_		
13 ± 3	17.5 ± 3	3>	50 ± 3	2.5>	0.21	14 ± 3	5400	2.62

**Table 2:** Physical properties of silica sand.

No.	Size (mm)	Density (20 °C)	F.M
6	≤0.3	2.62	1.95

**Table 3:** Properties of wollastonite mineral fiber.

Properties	Values
Appearance	White
Shape	Acicular
Length	0.4–0.6 mm
Transverse dimension	25~150 µm
Maximum aspect ratio	3~20
Coefficient of expansion (mm/mm/°C )	6.5 × 10^−6^
Density (g/mm^3^)	2.9
Water solubility (g/100 cc)	0.0095
pH	9.9

**Table 4:** Properties of SB latex polymer.

Solids Content (%)	Styrene Content (%)	Butadiene Content (%)	pH	Density (g/mm^3^)	Surface Tension (Dyne/cm)	Particle Size (Å)	Viscosity (cps)
49	34 ± 1.5	66 ± 1.5	11.0	1.02	30.57	1700	42

**Table 5:** Mix proportion.

Type of Mix	W */B (%)	Total Water	Unit Weight (kg/m^3^)						Flow Value (mm)
			Mixing Water	1:1.5 (Binder)		Wollastonite (B × 3%)	SB Latex Polymer (B × 5%)		
				Rapid Set Cement	Silica Sand		Solid	Water	
M0L0	15.18	304.00	304	801	1202	-	-	-	200
M0L5	12.05	245.02	193	813	1220	-	49.98	52.02	205
M3L0	16.09	299.00	299	743	1115	56	-	-	200
M3L5	14.75	268.41	222	728	1092	55	44.59	46.41	205

*: Total water (Mixing water + Water in latex.

## Data Availability

Not applicable.

## References

[1] Kim S. (2017). Development of Artificial Crack Testing Method for Injection Type Repair Materials Used in Leakage Cracks of Concrete Structure in an Underground Environment.

[2] Lee J. (2006). Experimental Study on the Characteristics of Penetrating Surface Protection Materials to Promote Concrete Structure Durability.

[3] Lee H. (2012). Performance Evaluation for Initial-Crack Decrease of Polymer Mortar with Jute Fiber.

[4] Korea Institute of Civil Engineering (2003). Development of Repair Rehabilitation Material Qualities and Methods Evaluation Standard of Reinforced Concrete Bridges.

[5] Kwon M., Seo H., Lim J., Kim J. (2013). The Properties of Durability and Strength of Fiber-Reinforced Polymer-Modified Mortars Using Eco-Friendly UM Resin. J. Korea Concr. Inst..

[6] Kong T. (2005). Enhanced Durability Performance of Regulated Set Cement Concrete.

[7] Mathur R., Misra A.K., Goel P. (2007). Influence of wollastonite on mechanical properties of concrete. J. Sci. Ind. Res..

[8] Soliman A.M., Nehdi M.L. (2012). Effect of Natural Wollastonite Microfibers on Early-Age Behavior of UHPC. J. Mater. Civ. Eng..

[9] Kumar R. Wollastonite Mineral Fibre in Manufacturing of an Economical Pavement Concrete. Proceedings of the Fourth International Conference on Sustainable Construction Materials and Technologies.

[10] Lee J. (2007). Development of High Early Strength Latex Modified Sprayed-Mortar.

[11] Park S. (2008). Properties of Latex Modified Concrete(LMC) by Binder Content and Effect on Chloride Ion Diffusion.

[12] Lee J. (2013). Target Performance Based Optimum Mix Design and Performance Evaluation of Recycled Aggregate and Industrial by Products Concrete.

[13] Han S. (2007). Evaluation on the Durability of the Concrete with Nano Level Ceramic Based Coatings.

[14] Kim H. (2007). Development and Assessment of High-Performance Shotcrete for Permanent Support.

[15] Lim J. (2017). A Study on the Development of High Durability Repair Materials for Emergency Maintenance of Port Facilities.

[16] Ministry of Construction & Transportation (2003). Development of Anti-Bacteria and Anti-Insect Concrete and Mortar Using Microcapsule.

[17] Kwon S., Nishiwaki T., Choi H., Mihashi H. (2015). Effect of Wollastonite Microfiberon Ultra-High-Performance Fiber-Reinforced Cement-Based Composites Based on Application of Multi-Scale Fiber-Reinforcement System. J. Adv. Technol..

